# Identification of genomic variants causing sperm abnormalities and reduced male fertility

**DOI:** 10.1016/j.anireprosci.2018.02.007

**Published:** 2018-02-10

**Authors:** Jeremy F. Taylor, Robert D. Schnabel, Peter Sutovsky

**Affiliations:** Division of Animal Sciences, University of Missouri, Columbia, MO, 65211, USA

**Keywords:** Bull fertility, Genomic selection, Genome-wide association study, Candidate genes, Quantitative trait loci, Nanopurification

## Abstract

Whole genome sequencing has identified millions of bovine genetic variants; however, there is currently little understanding about which variants affect male fertility. It is imperative that we begin to link detrimental genetic variants to sperm phenotypes *via* the analysis of semen samples and measurement of fertility for bulls with alternate genotypes. Artificial insemination (AI) bulls provide a useful model system because of extensive fertility records, measured as sire conception rates (SCR). Genetic variants with moderate to large effects on fertility can be identified by sequencing the genomes of fertile and subfertile or infertile sires identified with high or low SCR as adult AI bulls or yearling bulls that failed Breeding Soundness Evaluation. Variants enriched in frequency in the sequences of subfertile/infertile bulls, particularly those likely to result in the loss of protein function or predicted to be severely deleterious to genes involved in sperm protein structure and function, semen quality or sperm morphology can be designed onto genotyping assays for validation of their effects on fertility. High throughput conventional and image-based flow cytometry, proteomics and cell imaging can be used to establish the functional effects of variants on sperm phenotypes. Integrating the genetic, fertility and sperm phenotype data will accelerate biomarker discovery and validation, improve routine semen testing in bull studs and identify new targets for cost-efficient AI dose optimization approaches such as semen nanopurification. This will maximize semen output from genetically superior sires and will increase the fertility of cattle. Better understanding of the relationships between male genotype and sperm phenotype may also yield new diagnostic tools and treatments for human male and idiopathic infertility.

## 1. Introduction

The United States is among the world’s largest producers of beef and dairy products, two industries with a combined herd size of 93 million animals. The direct, retail value-based annual economic impact of the US cattle industry exceeds $74 billion (USDA, 2013). Improvements in the fertility of cows have been realized through optimized heat detection and timed artificial insemination (AI) but improvements in the reproductive rate of the national herd has been limited by a paucity of accurate predictors of bull fertility, including but not limited to heritable traits influencing semen quality, gonadal development and function (both testis and epididymis), testicular size and non-return/calving rate that have higher heritabilities than female reproductive traits (Berry et al., 2014). Improvements in sire conception rate (SCR) can only be achieved by recording fertility and sperm phenotypes on AI sires and utilizing whole-genome single nucleotide polymorphism (SNP) genotypes to select to improve SCR which will result in improvements in all fertility component traits including sperm quality and longevity in the female tract, as well as paternally influenced embryo traits that affect embryo cleavage, development to blastocyst and implantation. Due to titration of semen doses to achieve uniform fertility in high-demand Holstein bulls, SCR has a low heritability (Kuhn and Hutchison, 2008), and the inherent fertility of these sires may be indeterminable even if thousands of AI services are recorded (Amann and DeJarnette, 2012). Furthermore, polymorphisms in genes that contribute to variation in bull reproductive traits are yet to be discovered and validated but are required if we are to further improve on the current, primarily subjective, semen evaluation process with a biomarker-based sperm phenotyping system (Sutovsky et al., 2015).

While the creation of mouse knock-out or mutant models may reveal if a particular gene is essential for fertility, we can only gain an understanding of individual differences in fertility, particularly in livestock, from studying naturally occurring polymorphisms. This is particularly difficult to accomplish in human because there is no such thing as a man with extensive fertility records. However, large animal models provide a unique opportunity since, for example, most AI services and SCR are recorded for breeding bulls, enabling researchers to link fertility records to genotypes in thousands of bulls. One of the goals of such efforts is to link polymorphisms in genes controlling spermatogenesis, sperm function and pre-implantation embryo development to aberrant sperm phenotypes in bulls used in AI. Accessible animal model cohorts include presumed infertile young sires that failed Breeding Soundness Evaluation (BSE) as well as fertile sires in active AI with highly accurately estimated SCR phenotypes from which phenotypically extreme animals can be sampled for sequencing and genomic data mining. Such stakeholder driven genomic research projects benefit commercial agriculture, human assisted reproductive therapies and the fields of genetics and reproductive biology. Research outcomes have the potential to improve the fertility of the US and worldwide cattle herds through the optimization of selection for bull fertility, based on genomics and semen phenotyping for sperm functionality traits. The direct beneficiaries of research efforts are the stakeholders in all sectors of the beef and dairy cattle industries, but particularly AI organizations, who seek to reduce the number of infertile and subfertile bulls brought into AI, which is a profit-limiting factor.

Recently, Select Sires, one of the AI industry leaders, published a list of 36 Holstein AI-sires whose genomes harbor fertility-reducing haplotypes which are not observed in homozygous form in living animals; however, while these haplotypes are presumed to harbor early embryonic lethal mutations, the specific biological cause of reduced fertility associated with most of these multi-SNP haplotypes is not known (Collective, 2015). Improvements in male fertility in agriculture can undoubtedly be achieved through the implementation of genomic selection (García-Ruiz et al., 2016) and the optimization of AI based on genomics, biomarker-based fertility testing, technology transfer and research-driven infrastructure improvement. Through translational research, the development of diagnostics and treatments for human male and idiopathic infertility could also be advanced. Ultimately, intellectual gains from such research will result from a better understanding of the male genotype-to-sperm phenotype relationship.

## 2. Recent innovations in bovine genomics

A number of recently completed and ongoing sequencing projects have identified potential loss of function [LOF; premature stop codons and insertions/deletions (indels) causing frameshifts] and non-synonymous amino acid mutations in fertility-related genes (Charlier et al., 2016; Daetwyler et al., 2014). This knowledge makes it possible to link polymorphisms in fertility-associated genes with sperm phenotypes in males with extensive fertility records. In stark contrast to the “fishing expeditions” of the past, this hypothesis-driven approach is based on variant discovery achieved by whole genome re-sequencing, and genome-wide association analysis (GWAA) of identified mutations with SCR and sperm phenotypes. Due to the high use of AI and dominance of the breed within dairy production, this work is primarily conducted in the Holstein. The advantage of Holstein as a primary model is ease of access to AI-conception rate data, semen for phenotyping and existing BovineSNP50 genotype data (García-Ruiz et al., 2016; Matukumalli et al., 2009), as well as data from other types of genotyping chips. Consistent with the fact that SCR is heritable when semen is titrated to constant doses of post-thaw progressively motile spermatozoa (Taylor et al., 1985), as is semen quality (Berry et al., 2014), polymorphisms in genes encoding proteins previously implicated in sperm function and production have been identified. Relevant sperm phenotypes can be characterized by conventional flow cytometry or by state-of-the-art image-based flow cytometry (IBFC) (Buckman et al., 2009; Buckman et al., 2013). For the first time, it will be possible to systematically catalog and phenotype polymorphisms in genes relevant to bull sperm-function and embryo development, which will be made publically available through deposition in public polymorphism databases such as dbSNP. These efforts will pave the way for novel approaches to bull fertility testing and the improvement of AI conception rates through innovative, unconventional approaches such as semen nanopurification (Sutovsky and Kennedy, 2013; Odhiambo et al., 2014), aligning with the recently implemented USDA Program for Bioprocessing Engineering & Nanotechnology (USDA-NIFA, 2013; Feugang, 2017). Innovative genomic approaches are now being adopted by the industry through the development of variant discovery methodologies and resource populations (Van Tassell et al., 2008), and the development of genotyping assays such as the Illumina BovineSNP50 (Matukumalli et al., 2009) and BovineHD assays, the Affymetrix BOS1 assay and the GeneSeek suite of assays including the GGP-F250 (Collective, 2016), which have been extensively used by the US cattle industries (Cole et al., 2009; Rolf et al., 2010; Saatchi et al., 2011; VanRaden et al., 2009; Wiggans et al., 2009).

## 3. Rationale and need for a better understanding of bull fertility

Substantial progress has been achieved in the optimization of reproductive performance of cows and heifers (Leitman et al., 2009; Mallory et al., 2010, 2011). While some additional gains can be made by further improving female fertility (Johnson et al., 2011), the genetic correlation between male and female fertility is low (Hansen, 1979; Syrstad, 1981) and, consequently, maximizing the productivity of the national herd requires simultaneous improvement of the reproductive performance of bulls (Petrunkina and Harrison, 2011). Bull semen analysis is still performed using conventional microscopy-based methods, although new biomarkers and instrumentation have been introduced for the automated, objective, high-throughput analysis of semen (Hossain et al., 2011; Sutovsky and Lovercamp, 2010). Based on conventional andrology, yearling bulls with inferior sperm quality are simply eliminated and prevented from entering the AI pool following BSE; however, this culling practice does little towards reducing the frequency of defective recessive alleles, which exist primarily in heterozygous carrier females. Furthermore, low fertility in some bulls may be masked by the high sperm numbers (15 million or more) routinely used in AI doses. Due to the lack of large volumes of population-based phenotypic fertility data, it is unlikely that human or mouse research will identify genes in which polymorphisms cause subfertility or subtle changes in fertility phenotypes. Consequently, a livestock model in which thousands of individuals have conception rate data based on from hundreds to many thousands of inseminations is ideally suited to study the relationship between male fertility and genome-wide variation.

With an average AI conception rate of ~60%, considerable opportunity for the improvement of herd fertility will come from improvements in bull reproductive performance. This recognition has accelerated the adoption of genomic tools by AI industry stakeholders (Collective, 2015; García-Ruiz et al., 2016). Through genomic selection for male reproductive traits and sperm phenotyping, it would also be possible to increase the number of AI doses from high-demand bulls with superior production traits, further increasing selection intensity and rates of improvement in production traits. Some published evidence in bulls and males of other species already exists to support this approach. Polymorphisms in two bovine genes encoding sperm head proteins, integrin subunit beta 5 (*ITGB5*) and collagen type I alpha 2 chain (*COL1A2*), have been associated with variation in bull fertility (Feugang et al., 2009). Polymorphisms within protamine genes *PRM1* and *PRM2* have been associated with sperm quality in humans (Tuttelmann et al., 2009), and the extent of sperm protamination is correlated with the conception rates of AI sires (Dogan et al., 2015). A SNP in a gene involved in sperm head shaping, *SPATA1*, has been associated with the fertility of stallions (Giesecke et al., 2009). As expected, many spermatogenesis/sperm related LOF alleles are not embryonic lethals since the functions of their protein products in healthy individuals are restricted to germ cells and they are therefore not essential for life.

## 4. Approach to the identification of genes affecting bull fertility

The goal of collaborative research in our laboratories is to link defective sperm phenotypes prevalent in bulls with low SCR to mutations in individual genes and cellular pathways controlling spermatogenesis, sperm function and the paternal genome influence on early embryo development. Our approach is based on whole-genome sequencing for variant discovery, followed by large-scale genotyping of bulls phenotypically characterized as infertile based upon BSE or with acceptable but varied fertility (*i.e.,* bulls that passed BSE and that lie within opposing tails of the SCR distribution) to enable association analyses to establish phenotype-to-genotype associations. This approach allows the identification of mutations associated with high/low fertility and also the associated sperm phenotypes, including sperm morphology, sperm protein structure, function and localization. However, the process of whole genome sequencing frequently will reveal hundreds of thousands of variants that differ in allele frequency between low and high fertility bulls and these cannot all be cost-effectively genotyped in order to test genotype-to-phenotype associations in large samples of phenotyped individuals. We have participated in the development of the BovineSNP50, BovineHD, PorcineSNP60 and the bovine GGP-F250 (available through GeneSeek, Lincoln, NE) assays that query genotypes at from 54,000 to 777,000 SNP loci. These assays typically cost in the vicinity of $1M to develop (generating sufficient reagents to genotype ~10,000 individuals) and this is beyond the means of most small collaborative projects. However, small numbers of novel variants (up to 4–5,000) can cost effectively be genotyped as an “add-on” to the content of some of the assays commercialized by GeneSeek leading to per sample genotyping costs of $40–$60 enabling several thousand individuals to be genotyped within the budget of R01 or USDA NIFA grants.

Of course, this requires a prioritization or filtering of the variants discovered in the sequencing to those likely to impact genes associated with sperm function. To enable this, we propose to select polymorphisms in candidate genes based on the following criteria: (1) Identified mutation causes a predicted loss of function allele, predicted isoform or non-synonymous amino acid sequence change, (2) Mutation is at a moderate frequency (*i.e.*,>1%) in the U.S. Holstein population, as estimated using available sequence data for ~500 Holsteins including the 1000 Bull Genomes Project data (Daetwyler et al., 2014), (3) Gene is essential for life or orthologous gene KO mice are infertile or variation in the human ortholog has been associated with infertility (information available from mouse and human databases; *e.g*., JAX ReproGenomics mouse database and the 1000 Human Genomes database). Detected mutations can be characterized in a number of ways, including whether the mutation is located within an annotated gene or a predicted regulatory region, and is likely to change the function of a gene *via* an amino acid substitution, a premature stop codon, frameshift or deletion. Mutations creating amino acid substitutions can be analyzed using SIFT (Kumar et al., 2009) to detect those that are predicted to damage protein function. Based on the sequences of 234 taurine animals used in the design of the GGP-F250 assay, we anticipate that this approach will yield~180,000 protein coding variants with a minor allele frequency≥0.01, observed in ≥2 sequenced individuals, of which>16,000 will be predicted by SIFT to have severely deleterious effects on protein function. We will prioritize the selection of polymorphisms within genes with known function in male reproductive processes, including those that control post-meiotic differentiation of spermatids, genes regulating the mitotic/meiotic phases of spermatogenesis and those in control of male reproductive tract development. Among these are the genes within the ubiquitin-proteasome system, which is essential for protein turnover during spermatogenesis, as documented in male-infertile mouse mutants (Hermo et al., 2009; Sutovsky, 2011). Gene expression in members of the ubiquitin-proteasome system is dysregulated in the testes of infertile men (Platts et al., 2007).

In addition to LOF and amino acid substitution-causing polymorphisms, functionally impaired alleles that are in moderate frequency in cattle and therefore have the greatest impact on fertility will include polymorphisms in non-coding regions, particularly the promoter and untranslated regions of genes and within regulatory regions. Since the regions of the bovine genome that are involved in the regulation of gene function are almost completely unknown, the only way that we can potentially identify regulatory polymorphisms is to identify polymorphisms that lie with regions of the genome that are evolutionarily conserved among those ruminant species that have reference genome sequences. These regions of the genome are postulated to be conserved in sequence between species, because they have regulatory function and consequently strong purifying selection operates on these regions to maintain the integrity of the underlying sequence.

The genotyped sires are presumed to differ in their genetic merit for SCR because they individually harbor different numbers of deleterious alleles and/or alleles that differ in their severity of effect on fertility. Recessive lethal alleles that impact fertility *via* embryonic viability impact both male and female fertility and so are included in the variant filtering/selection process. From this process, polymorphisms can be prioritized for their inclusion in the design of custom SNP genotyping assays, to genotype sires with the highest and lowest SCR phenotypes, and also sires that historically failed BSE and that did not enter active AI service. The produced genotypes will be merged with any available BovineSNP50 and other chip genotypes and used to perform GWAA analysis on SCR and sperm phenotypes determined from the cryopreserved semen of these bulls.

This approach identifies variants that are associated with male fertility or sperm phenotypes, which does not imply their causality. Establishing the causality of a DNA variant is analogous to presenting compounded evidence in a criminal trial to conclude guilt beyond a reasonable doubt. If a variant can be shown to have a functional effect on gene expression or the protein encoded by that gene and if the variant is the most strongly associated with a trait of all tested variants in the genomic region in several independent replicated studies, we might consider this to be sufficient evidence of causality. For the purpose of producing genomic estimates of genetic merit, there is no need to establish or even rely upon the causality of effect of a variant. All that is required is that the alleles at the variant consistently predict the animals with enhanced fertility across different populations (same breed in different countries or different breeds) of cattle. This requires the presence of very strong linkage disequilibrium (correlation) between the alleles at the variant and the alleles at the causal variant, and of course, this relationship is perfect when the tested variant is the causal variant. To establish the predictive capability of variants identified as trait-associated in a GWAA, the simplest strategy is to include these variants on the genotyping assays that are now being widely used in the cattle industries to enable the genotyping of hundreds of thousands of animals from different breeds. If the variants consistently predict high SCR bulls using AI data recorded for Holsteins, Jerseys, and Norwegian Red dairy cattle and U.S. Angus and German Fleckvieh cattle, we can conclude that the variant is very likely causal.

## 5. Linking genetic variants with sperm protein phenotypes measurable in semen

A deleterious non-synonymous substitution or LOF mutation in a gene controlling spermatogenesis or sperm function could render the carrier sire and some of its male offspring subfertile *via* a change in the quantity, localization and function of the sperm protein encoded by this gene. By sequencing the genomes of subfertile bulls with sperm phenotypes characterized by cell imaging, proteomic analysis and most conveniently by using high throughput multiplex flow cytometry candidate variants can be identified that underlie abnormal phenotypes. Ultimately, by sequencing sufficient numbers of bulls with aberrant sperm phenotypes, identification of variants that are enriched in frequency in these animals relative to bulls with normal sperm phenotypes will elucidate the risk variants that are responsible for morphological sperm defects. Sperm biomarkers can be directly linked to sperm phenotypes using IBFC of spermatozoa labeled with fluorescently conjugated antibodies against target biomarkers (Buckman et al., 2009). Combining high throughput flow cytometry with rapid multi-channel image acquisition, the relative fluorescence of a target allele-encoded protein can be directly linked to its localization and fluorescence pattern, and also potentially associated with a morphological sperm phenotype on output IBFC image galleries of spermatozoa acquired simultaneously with bright field and epifluorescence imaging.

To validate a new biomarker, multiplex flow cytometry of previously validated sperm quality biomarkers (Kennedy et al., 2014; Sutovsky et al., 2002, 2007; Odhiambo et al., 2011) can be applied, and the flow cytometric outputs correlated with fertility records using established statistical tools (Kennedy et al., 2014; Odhiambo et al., 2011). Illustrating this approach, IBFC revealed the absence of fertilization-associated WBP2NL (syn. PAWP) protein from the spermatozoa of bulls with grossly malformed sperm heads and revealed a correlation with sperm-defect associated proteins such as ubiquitin, and with abnormal patterns of sperm labeling with lectins PNA and LCA (Kennedy et al., 2014). The unsurpassed accuracy of multiplex IBFC can be used to enhance the conventional semen analysis used in the AI industry to identify sperm phenotypes caused by male infertility alleles. With regard to sperm surface proteins and lectin ligands, identifying molecular targets on the defective sperm surface will allow the improvement of semen nanopurification technologies (Odhiambo et al., 2014). In addition to using genome-based biomarker discovery approaches, nanopurification or other methods (sperm swim-up, gradient centrifugation, magnetic-activated cell sorting) can be used to enrich samples for defective spermatozoa, and proteomic analyses (mass spectrometry) can be used to identify proteins that are enriched in the defective sperm fractions. For example, seminal plasma-derived binder of sperm protein BSP5 (Hung and Suarez, 2012) was found to be enriched in the defective sperm fraction after nanopurification (Odhiambo et al., 2014). Alternative splicing of transcripts and posttranslational modifications to proteins are also important molecular mechanisms that may underlie variation in fertility since they can alter the size and activity of proteins. These alterations can be detected by RNA-seq or mass spectrometry analyses of RNA or proteins extracted from sperm samples.

## 6. Conclusions and perspectives

Whole genome sequencing has revealed that there are more variants present in the bovine genome than in the human genome reflecting the difference in age of the two species. We estimate that over 3000 cattle genomes have now been sequenced worldwide revealing over 100 million polymorphisms. dbSNP build 151 contains over 104 million entries for *Bos taurus* and over 17 million entries for *Bos indicus*. Some of these variants create variation in male fertility, however, the specific polymorphisms that affect sperm phenotypes and fertilizing ability, or that cause early embryo loss, are generally unknown. Assuming that polymorphisms in paternally- inherited, fertility associated genes expressed during spermatogenesis and preimplantation embryo development are responsible for male subfertility or infertility, and possibly also for early pregnancy loss, the tools are now available to allow us to link detrimental genetic variants to sperm phenotypes measurable in an individual semen sample that impact male fertility *in vivo* and *in vitro*.

Artificial insemination bulls provide a very useful model system for this purpose because their DNA is publically accessible, they have unparalleled, extensive fertility records from thousands of AI services, and their fertility *in vivo*, measured by sire conception rate, correlates to some extent with sperm phenotypes, particularly when sperm phenotyping is biomarker-based. To identify genetic differences between fertile and subfertile or infertile sires, whole-genome sequencing can now be inexpensively used to identify polymorphisms with alleles that are differentially enriched in high *versus* low SCR bulls, or in fertile bulls *versus* yearling bulls that failed BSE. Assuming that fertile and infertile bulls fall on the opposite ends of the fertility spectrum due to polymorphisms in relatively few fertility-associated genes, these genes, and the responsible functional polymorphisms, can be revealed by sequencing relatively few infertile bulls. Likewise, by sequencing bulls with high *versus* low SCR and identifying polymorphisms with different alleles enriched between the two groups, alleles which influence semen fertility *via* semen quality or sperm morphology can also be discovered. Using the discovered polymorphisms, genotyping assays that are routinely used in the beef and dairy industries that undergo periodic redesigns can be redesigned to incorporate polymorphisms expected to impact sperm protein structure and function, as well as SCR variation. To link mutations influencing sire fertility to sperm phenotypes easily measurable in semen samples, high throughput multiplex flow cytometry, proteomics and cell imaging can be used to validate these variants as sperm biomarkers. In addition to polymorphisms in protein coding sequences, attention should also be paid to those encoding sperm-borne small non-coding RNAs that could impact early embryo development.

This approach assumes that deleterious mutations in sperm-relevant genes will alter sperm phenotype and change the quantity, localization and function of sperm proteins, thus influencing a carrier’s fertility. Our approach at this stage is necessarily gene-centric due to the lack of annotation of regulatory regions within the bovine genome. However, mutations within sncRNAs and their targets as well as regulatory regions are likely to be even more important for quantitative trait variation than are mutations within gene coding regions and should be prioritized for future research as these genomic regions are elucidated. The focus on genetic control of bull fertility by no means diminishes the importance of epigenetic factors and natural fluctuations in sperm quality that are due to bull age or seasonal influences. Integrating the genome-wide polymorphisms and AI fertility records with sperm phenotypes and biomarker expression patterns will accelerate biomarker discovery and validation, improve routine semen testing in bull studs and identify new targets for cost-efficient AI dose optimization approaches such as semen nanopurification. This approach will also support industry efforts to maximize AI dose output from valuable sires and does not disregard the value of information provided by conventional semen analysis. As an example of the utility of this approach, nanopurification targeting aberrant sperm surface ligands made it possible to obtain full AI dose-level pregnancy rates when half-AI-doses of nanopurified spermatozoa were used, with no adverse effects in 798 inseminated cows, which gave birth to 466 healthy calves (Odhiambo et al., 2014). The technique has already been adapted for boars (Feugang et al., 2015), where it increased juvenile offspring weight and carcass quality. In addition to improving the fertility of the U.S. cattle herd through the selection of AI bulls for sire fertility based on predictive genomic variants and improvements in semen evaluation, advances in genomics and sperm phenotyping may also yield new diagnostic tools and treatments for human male and idiopathic infertility. This avenue of research should also lead to a better understanding of the relationships between genotype, sperm phenotype and male fertility.
